# Tetra­kis(di­propyl­ammonium) tetra­kis(oxa­lato-κ^2^ *O* ^1^,*O* ^2^)stannate(IV) mono­hydrate: a complex with an eight-coordinate Sn^IV^ atom

**DOI:** 10.1107/S160053681303496X

**Published:** 2014-01-18

**Authors:** Ndongo Gueye, Libasse Diop, Helen Stoeckli-Evans

**Affiliations:** a Laboratoire de Chimie Minerale et Analytique, Departement de Chimie, Faculte des Sciences et Techniques, Universite Cheikh Anta Diop, Dakar, Senegal; b Institute of Physics, University of Neuchâtel, rue Emile-Argand 11, CH-2000 Neuchâtel, Switzerland

## Abstract

In the title salt, [(CH_3_CH_2_CH_2_)_2_NH_2_]_4_[Sn(C_2_O_4_)_4_]·H_2_O, the Sn^IV^ atom of the stannate anion is located on a special position with -42*m* symmetry. It is eight-coordinated by four chelating oxalate anions. The di­propyl­ammonium cation possesses mirror symmetry while the lattice water mol­ecule is disordered about a position with -42*m* symmetry and has an occupancy of 0.25. In the crystal, the anions and cations are linked by N—H⋯O hydrogen bonds, forming a three-dimensional network. This network is futher stabilized by weak O—H⋯O hydrogen bonds involving the water mol­ecules and oxalate O atoms. The crystal studied was refined as an inversion twin.

## Related literature   

For the chemistry of organotin complexes, see: Evans & Karpel (1985 ▶). For examples of zirconate anions with eight-coordinate Zr^IV^ atoms, see: Fu *et al.* (2005 ▶); Imaz *et al.* (2007 ▶). For an example of a related oxalatostannate(IV) complex, see: Gueye *et al.* (2010 ▶).
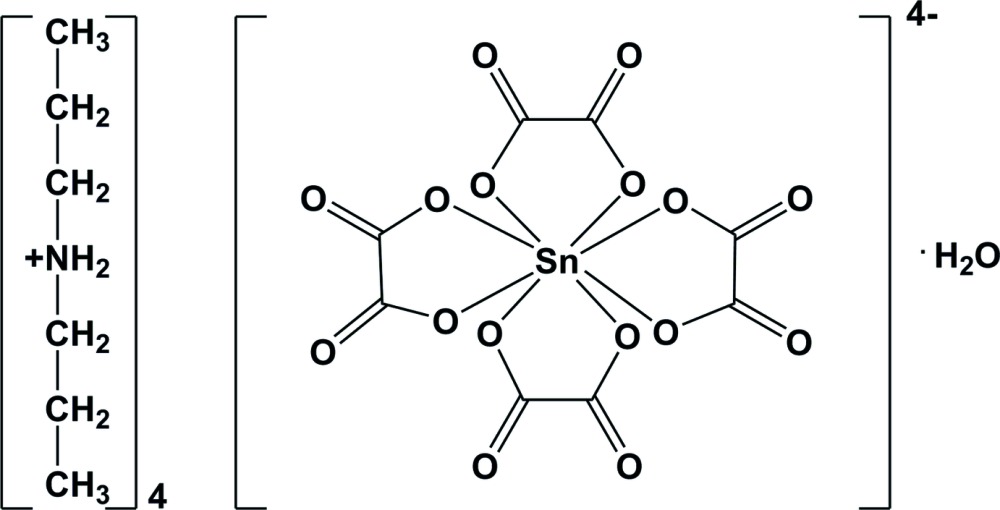


## Experimental   

### 

#### Crystal data  

(C_6_H_16_N)_4_[Sn(C_2_O_4_)_4_]·H_2_O
*M* *_r_* = 897.57
Tetragonal, 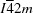

*a* = 14.5996 (6) Å
*c* = 9.5718 (4) Å
*V* = 2040.21 (19) Å^3^
*Z* = 2
Mo *K*α radiation
μ = 0.70 mm^−1^
*T* = 173 K
0.45 × 0.30 × 0.22 mm

#### Data collection  

STOE IPDS2 diffractometer
Absorption correction: multi-scan (*MULscanABS* in *PLATON*; Spek, 2009 ▶)’ *T*_min_ = 0.540, *T*_max_ = 1.000
9636 measured reflections
1030 independent reflections
1027 reflections with *I* > 2σ(*I*)
*R*_int_ = 0.071

#### Refinement  

*R*[*F* ^2^ > 2σ(*F* ^2^)] = 0.021
*wR*(*F* ^2^) = 0.054
*S* = 1.08
1030 reflections
83 parameters
3 restraints
H atoms treated by a mixture of independent and constrained refinement
Δρ_max_ = 0.39 e Å^−3^
Δρ_min_ = −0.62 e Å^−3^
Absolute structure: Refined as an inversion twin
Absolute structure parameter: 0.45 (4)

### 

Data collection: *X-AREA* (Stoe & Cie, 2009 ▶); cell refinement: *X-AREA* (Stoe & Cie, 2009 ▶); data reduction: *X-RED32* (Stoe & Cie, 2009 ▶); program(s) used to solve structure: *SHELXS2013* (Sheldrick, 2008 ▶); program(s) used to refine structure: *SHELXL2013* (Sheldrick, 2008 ▶); molecular graphics: *PLATON* (Spek, 2009 ▶) and *Mercury* (Macrae *et al.*, 2006 ▶); software used to prepare material for publication: *SHELXL2013* (Sheldrick, 2008 ▶), *PLATON* (Spek, 2009 ▶) and *publCIF* (Westrip, 2010 ▶).

## Supplementary Material

Crystal structure: contains datablock(s) I, global. DOI: 10.1107/S160053681303496X/wm2795sup1.cif

Structure factors: contains datablock(s) I. DOI: 10.1107/S160053681303496X/wm2795Isup2.hkl

CCDC reference:

Additional supporting information:  crystallographic information; 3D view; checkCIF report

## Figures and Tables

**Table 1:** Hydrogen-bond geometry (Å, °)

*D*—H⋯*A*	*D*—H	H⋯*A*	*D*⋯*A*	*D*—H⋯*A*
N1—H1*AN*⋯O1^i^	0.88 (3)	2.50 (5)	3.091 (6)	125 (5)
N1—H1*AN*⋯O2^i^	0.88 (3)	2.12 (3)	2.997 (8)	179 (6)
N1—H1*BN*⋯O2	0.88 (3)	2.02 (4)	2.821 (7)	151 (5)
N1—H1*BN*⋯O4	0.88 (3)	2.44 (5)	3.105 (6)	133 (5)
O1*W*—H1*WA*⋯O3^ii^	0.85	2.27	3.125 (7)	179

Symmetry codes: (i) 
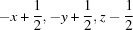
; (ii) 
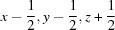
.

**Fig. 1.**
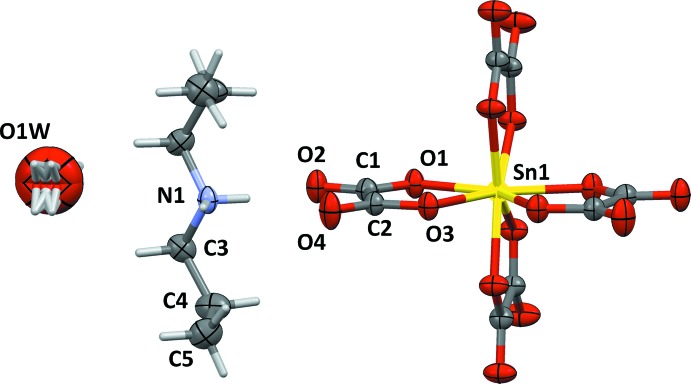
A view of the molecular components of the title salt. Displacement ellipsoids are drawn at the 50% probability level.

**Fig. 2.**
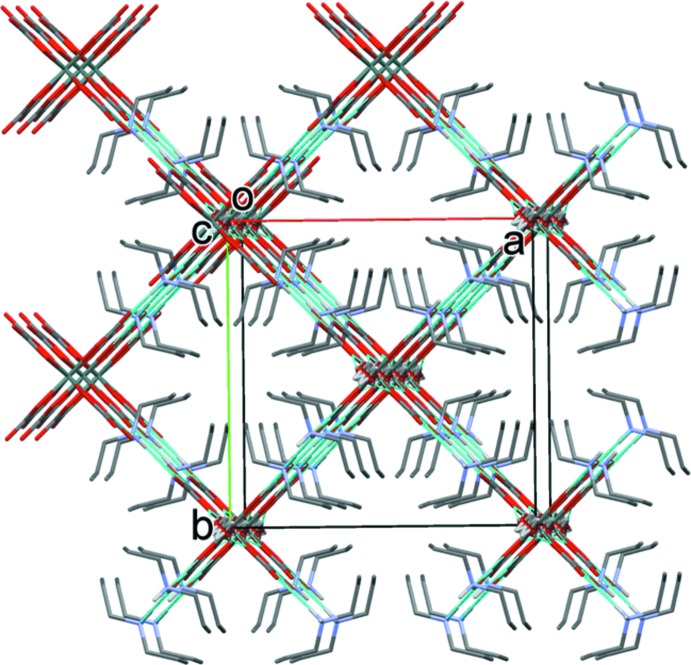
A view approximately along the c axis of the crystal packing of the title compound. The hydrogen bonds are shown as dashed lines (see Table 1 for details; C-bound H atoms have been omitted for clarity).

## supplementary crystallographic information

### 1. Comment

The chemistry and applications of organotin(IV) complexes have been extensively discussed by Evans & Karpel (1985). Continuing our interest in Sn^IV^ oxalate complexes (Gueye *et al.*, 2010), we studied the reaction of dipropylammonium oxalate with SnBr_2_, and report herein on the crystal structure of the title compound, ((CH_3_CH_2_CH_2_)_2_NH_2_)_4_[Sn(C_2_O_4_)_4_]^.^H_2_O.

The molecular structure of the title salt is illustrated in Fig. 1. The Sn^IV^ atom of the stannate anion is located on a special position with 42*m* symmetry. It is chelated by four bidentate oxalate ions, each lying in a mirror plane, and hence has a coordination number of eight. This coordination number has also been reported for some zirconium complexes, *viz*. bis(4,4'-bipyridinium) tetrakis(oxalato-κ^2^O,O')zirconate(IV) (Fu *et al.*, 2005), or several salts with general composition [(H_2_amine)_2_Zr(C_2_O_4_)_4_] (Imaz *et al.*, 2007).

The Sn1—O1 and Sn1—O3 bond lengths in the anion of the title compound are 2.142 (3) and 2.226 (3) Å, respectively. These values are similar to the bond lengths [2.189 (2) and 2.229 (2) Å] observed for another tin(IV)-oxalato complex, bis(dicyclohexylammonium)µ-oxalato-κ^4^O1,O2:O1',O2'-bis[aqua(oxalato -κ^2^O1,O2)diphenylstannate(IV)] (Gueye *et al.*, 2010).

In the crystal of the title salt, the stannate(IV) anions are linked *via* N—H···O hydrogen bonds to the [(CH_3_CH_2_CH_2_)_2_NH_2_]^+^ cations (which have mirror symmetry), forming a three-dimensional network. The water molecule (disordered about a position with 42*m* symmetry), is also involved in weak O—H···O hydrogen bonds with the stannate(IV) anions, hence futher stabilizing the three-dimensional network (Fig. 2).

### 2. Experimental

The title compound was prepared by reacting in a 1:1 molar ratio of SnBr_2_ and (Pr_2_NH_2_)_2_·C_2_O_4_ in methanol. The solution was allowed to stand and yielded colourless block-like crystals of the title compound. The proof of the presence of the tin atom was confirmed by the structure analysis and by electron dispersive X-ray (EDX) analysis.

### 3. Refinement

The NH_2_ and water H atoms were located in difference Fourier maps and refined with distance restraints: N—H =0.88 (3) Å and O—H = 0.85 Å, with *U*_iso_(H) = 1.2*U*_eq_(N) and = 1.5*U*_eq_(O), respectively. The C-bond H-atoms were included in calculated positions and treated as riding atoms: C–H = 0.99 and 0.98 Å for CH_2_ and CH_3_ H atoms, respectively, with *U*_iso_(H) = 1.5*U*_eq_(*C*-methyl) and = 1.2*U*_eq_(C) for other H-atoms.

### Crystal data

**Table:**

(C_6_H_16_N)_4_[Sn(C_2_O_4_)_4_]·H_2_O	*D*_x_ = 1.461 Mg m^−^^3^
*M**_r_* = 897.57	Mo *K*α radiation, λ = 0.71073 Å
Tetragonal, *I*42*m*	Cell parameters from 16495 reflections
*a* = 14.5996 (6) Å	θ = 2.0–26.1°
*c* = 9.5718 (4) Å	µ = 0.70 mm^−^^1^
*V* = 2040.21 (19) Å^3^	*T* = 173 K
*Z* = 2	Rod, colourless
*F*(000) = 944	0.45 × 0.30 × 0.22 mm

### Data collection

**Table:**

STOE IPDS2 diffractometer	1030 independent reflections
Radiation source: fine-focus sealed tube	1027 reflections with *I* > 2σ(*I*)
Plane graphite monochromator	*R*_int_ = 0.071
φ + ω scans	θ_max_ = 25.6°, θ_min_ = 2.0°
Absorption correction: multi-scan (*MULscanABS* in *PLATON*; Spek, 2009)'	*h* = −17→17
*T*_min_ = 0.540, *T*_max_ = 1.000	*k* = −17→17
9636 measured reflections	*l* = −10→11

### Refinement

**Table:**

Refinement on *F*^2^	Secondary atom site location: inferred from neighbouring sites
Least-squares matrix: full	Hydrogen site location: inferred from neighbouring sites
*R*[*F*^2^ > 2σ(*F*^2^)] = 0.021	H atoms treated by a mixture of independent and constrained refinement
*wR*(*F*^2^) = 0.054	*w* = 1/[σ^2^(*F*_o_^2^) + (0.030*P*)^2^ + 1.5457*P*] where *P* = (*F*_o_^2^ + 2*F*_c_^2^)/3
*S* = 1.08	(Δ/σ)_max_ < 0.001
1030 reflections	Δρ_max_ = 0.39 e Å^−^^3^
83 parameters	Δρ_min_ = −0.62 e Å^−^^3^
3 restraints	Absolute structure: Refined as an inversion twin
Primary atom site location: difference Fourier map	Absolute structure parameter: 0.45 (4)

### Special details

**Table:**

Geometry. All e.s.d.'s (except the e.s.d. in the dihedral angle between two l.s. planes) are estimated using the full covariance matrix. The cell e.s.d.'s are taken into account individually in the estimation of e.s.d.'s in distances, angles and torsion angles; correlations between e.s.d.'s in cell parameters are only used when they are defined by crystal symmetry. An approximate (isotropic) treatment of cell e.s.d.'s is used for estimating e.s.d.'s involving l.s. planes.
Refinement. Refined as a 2-component inversion twin.

### Fractional atomic coordinates and isotropic or equivalent isotropic displacement parameters (Å^2^)

**Table:**

	*x*	*y*	*z*	*U*_iso_*/*U*_eq_	Occ. (<1)
Sn1	0.5000	0.5000	0.5000	0.01816 (16)	
O1	0.40111 (16)	0.40111 (16)	0.5675 (4)	0.0298 (8)	
O2	0.29743 (14)	0.29743 (14)	0.5079 (8)	0.0356 (7)	
O3	0.43891 (16)	0.43891 (16)	0.3084 (3)	0.0281 (7)	
O4	0.3366 (2)	0.3366 (2)	0.2305 (4)	0.0436 (10)	
C1	0.3559 (2)	0.3559 (2)	0.4778 (6)	0.0276 (13)	
C2	0.3776 (2)	0.3776 (2)	0.3231 (5)	0.0249 (9)	
N1	0.1921 (2)	0.1921 (2)	0.3202 (5)	0.0289 (9)	
H1AN	0.195 (3)	0.195 (3)	0.228 (3)	0.035*	
H1BN	0.2322 (15)	0.2322 (15)	0.350 (6)	0.035*	
C3	0.0985 (3)	0.2174 (3)	0.3686 (4)	0.0369 (8)	
H3A	0.0947	0.2087	0.4711	0.044*	
H3B	0.0532	0.1760	0.3245	0.044*	
C4	0.0741 (3)	0.3159 (3)	0.3335 (4)	0.0475 (10)	
H4A	0.0160	0.3315	0.3811	0.057*	
H4B	0.1223	0.3564	0.3720	0.057*	
C5	0.0637 (3)	0.3361 (3)	0.1801 (4)	0.0497 (10)	
H5A	0.1232	0.3296	0.1337	0.075*	
H5B	0.0413	0.3989	0.1679	0.075*	
H5C	0.0199	0.2931	0.1388	0.075*	
O1W	0.0000	−0.026 (3)	0.5000	0.091 (15)	0.25
H1WA	−0.0167	−0.0355	0.5840	0.136*	0.25

### Atomic displacement parameters (Å^2^)

**Table:**

	*U*^11^	*U*^22^	*U*^33^	*U*^12^	*U*^13^	*U*^23^
Sn1	0.02109 (19)	0.02109 (19)	0.0123 (2)	0.000	0.000	0.000
O1	0.0352 (12)	0.0352 (12)	0.0190 (15)	−0.0117 (16)	−0.0020 (10)	−0.0020 (10)
O2	0.0417 (10)	0.0417 (10)	0.0234 (17)	−0.0187 (13)	−0.0001 (18)	−0.0001 (18)
O3	0.0346 (11)	0.0346 (11)	0.0151 (14)	−0.0089 (14)	−0.0026 (9)	−0.0026 (9)
O4	0.0548 (17)	0.0548 (17)	0.0211 (19)	−0.026 (2)	−0.0044 (12)	−0.0044 (12)
C1	0.0298 (13)	0.0298 (13)	0.023 (4)	−0.0010 (17)	−0.0020 (15)	−0.0020 (15)
C2	0.0273 (14)	0.0273 (14)	0.020 (2)	−0.0032 (18)	−0.0006 (13)	−0.0006 (13)
N1	0.0323 (14)	0.0323 (14)	0.022 (2)	−0.0083 (18)	−0.0029 (13)	−0.0029 (13)
C3	0.0356 (19)	0.049 (2)	0.0262 (17)	0.0009 (16)	0.0026 (14)	0.0014 (16)
C4	0.056 (3)	0.054 (3)	0.033 (2)	0.015 (2)	−0.0008 (19)	−0.0046 (18)
C5	0.064 (3)	0.051 (3)	0.035 (2)	0.002 (2)	0.000 (2)	0.0054 (18)
O1W	0.15 (5)	0.065 (15)	0.057 (7)	0.000	0.01 (3)	0.000

### Geometric parameters (Å, º)

**Table:**

Sn1—O1^i^	2.142 (3)	N1—H1AN	0.88 (3)
Sn1—O1^ii^	2.142 (3)	N1—H1BN	0.88 (3)
Sn1—O1^iii^	2.142 (3)	C3—C4	1.519 (6)
Sn1—O1	2.142 (3)	C3—H3A	0.9900
Sn1—O3	2.226 (3)	C3—H3B	0.9900
Sn1—O3^ii^	2.226 (3)	C4—C5	1.506 (5)
Sn1—O3^iii^	2.226 (3)	C4—H4A	0.9900
Sn1—O3^i^	2.226 (3)	C4—H4B	0.9900
O1—C1	1.269 (6)	C5—H5A	0.9800
O2—C1	1.240 (6)	C5—H5B	0.9800
O3—C2	1.274 (6)	C5—H5C	0.9800
O4—C2	1.225 (6)	O1W—O1W^v^	0.55 (6)
C1—C2	1.547 (7)	O1W—O1W^vi^	0.55 (6)
N1—C3	1.490 (4)	O1W—O1W^vii^	0.77 (8)
N1—C3^iv^	1.490 (4)	O1W—H1WA	0.8505
			
O1^i^—Sn1—O1^ii^	95.23 (5)	O4—C2—O3	127.3 (4)
O1^i^—Sn1—O1^iii^	95.23 (5)	O4—C2—C1	119.5 (4)
O1^ii^—Sn1—O1^iii^	144.86 (18)	O3—C2—C1	113.2 (4)
O1^i^—Sn1—O1	144.86 (18)	C3—N1—C3^iv^	111.0 (4)
O1^ii^—Sn1—O1	95.23 (5)	C3—N1—H1AN	110 (2)
O1^iii^—Sn1—O1	95.23 (5)	C3^iv^—N1—H1AN	110 (2)
O1^i^—Sn1—O3	142.09 (12)	C3—N1—H1BN	110.1 (19)
O1^ii^—Sn1—O3	75.60 (8)	C3^iv^—N1—H1BN	110.1 (19)
O1^iii^—Sn1—O3	75.60 (8)	H1AN—N1—H1BN	106 (6)
O1—Sn1—O3	73.05 (13)	N1—C3—C4	112.4 (3)
O1^i^—Sn1—O3^ii^	75.60 (8)	N1—C3—H3A	109.1
O1^ii^—Sn1—O3^ii^	73.05 (13)	C4—C3—H3A	109.1
O1^iii^—Sn1—O3^ii^	142.09 (12)	N1—C3—H3B	109.1
O1—Sn1—O3^ii^	75.60 (8)	C4—C3—H3B	109.1
O3—Sn1—O3^ii^	132.75 (11)	H3A—C3—H3B	107.9
O1^i^—Sn1—O3^iii^	75.60 (8)	C5—C4—C3	115.2 (4)
O1^ii^—Sn1—O3^iii^	142.09 (12)	C5—C4—H4A	108.5
O1^iii^—Sn1—O3^iii^	73.05 (13)	C3—C4—H4A	108.5
O1—Sn1—O3^iii^	75.60 (8)	C5—C4—H4B	108.5
O3—Sn1—O3^iii^	132.75 (11)	C3—C4—H4B	108.5
O3^ii^—Sn1—O3^iii^	69.05 (17)	H4A—C4—H4B	107.5
O1^i^—Sn1—O3^i^	73.05 (13)	C4—C5—H5A	109.5
O1^ii^—Sn1—O3^i^	75.60 (8)	C4—C5—H5B	109.5
O1^iii^—Sn1—O3^i^	75.60 (8)	H5A—C5—H5B	109.5
O1—Sn1—O3^i^	142.09 (12)	C4—C5—H5C	109.5
O3—Sn1—O3^i^	69.05 (17)	H5A—C5—H5C	109.5
O3^ii^—Sn1—O3^i^	132.75 (11)	H5B—C5—H5C	109.5
O3^iii^—Sn1—O3^i^	132.75 (11)	O1W^v^—O1W—O1W^vi^	90.004 (7)
C1—O1—Sn1	119.8 (3)	O1W^v^—O1W—O1W^vii^	45.002 (4)
C2—O3—Sn1	118.2 (3)	O1W^vi^—O1W—O1W^vii^	45.002 (4)
O2—C1—O1	124.0 (6)	O1W^v^—O1W—H1WA	108.2
O2—C1—C2	120.3 (5)	O1W^vi^—O1W—H1WA	84.7
O1—C1—C2	115.7 (4)	O1W^vii^—O1W—H1WA	98.9
			
Sn1—O1—C1—O2	180.000 (1)	O1—C1—C2—O4	180.000 (1)
Sn1—O1—C1—C2	0.000 (1)	O2—C1—C2—O3	180.000 (1)
Sn1—O3—C2—O4	180.000 (1)	O1—C1—C2—O3	0.000 (1)
Sn1—O3—C2—C1	0.000 (1)	C3^iv^—N1—C3—C4	174.3 (3)
O2—C1—C2—O4	0.000 (1)	N1—C3—C4—C5	67.2 (5)

Symmetry codes: (i) −*x*+1, −*y*+1, *z*; (ii) −*x*+1, *y*, −*z*+1; (iii) *x*, −*y*+1, −*z*+1; (iv) *y*, *x*, *z*; (v) −*y*, −*x*, *z*; (vi) *y*, −*x*, −*z*+1; (vii) *x*, −*y*, −*z*+1.

### Hydrogen-bond geometry (Å, º)

**Table:**

*D*—H···*A*	*D*—H	H···*A*	*D*···*A*	*D*—H···*A*
N1—H1*AN*···O1^viii^	0.88 (3)	2.50 (5)	3.091 (6)	125 (5)
N1—H1*AN*···O2^viii^	0.88 (3)	2.12 (3)	2.997 (8)	179 (6)
N1—H1*BN*···O2	0.88 (3)	2.02 (4)	2.821 (7)	151 (5)
N1—H1*BN*···O4	0.88 (3)	2.44 (5)	3.105 (6)	133 (5)
O1*W*—H1*WA*···O3^ix^	0.85	2.27	3.125 (7)	179

Symmetry codes: (viii) −*x*+1/2, −*y*+1/2, *z*−1/2; (ix) *x*−1/2, *y*−1/2, *z*+1/2.
